# Retraction: Methylation-induced downregulation and tumor suppressive role of microRNA-29b in gastric cancer through targeting LASP1

**DOI:** 10.18632/oncotarget.28381

**Published:** 2023-03-11

**Authors:** Hui Li, Guoqing Liu, Ke Pan, Xiongying Miao, Yong Xie

**Affiliations:** ^1^Department of Anesthesia, Second Xiangya Hospital, Central South University, Changsha, Hunan, China; ^2^Department of General Surgery, Second Xiangya Hospital, Central South University, Changsha, Hunan, China

**This article has been retracted:** In Figures 3A, 8D, and 7D, multiple wound healing assay images of gastric cancer cell lines overlap with the images from another published paper studying the effect of different miRNA in liver cancer [1], which was retracted. Figure 3A images of AGS cells transfected with miR-NC (AGS/miR-NC) and BGC-823 cells transfected with miR-29b (BGC-823/miR-29b) at 0 h time point overlap with the images of HEPG2/miR-137 and /miR-NC from Figure 2E of [1]; image of BGC-823/miR-29b+LASP1 from Figure 8D overlaps with HEPG2/miR-NC image of Figure 2E [1]; Figure 7D images of AGS/NC-siRNA at 0 h time point, AGS/LASP-siRNA and BGC-823/NC-siRNA at 48 h time point overlap with images of Figure 6E [1] HepG2/miR-137+EZH2 (0 h), HEPG2/miR-137 (24 h) and HEPG2/miR-137+EZH2 (24 h), respectively.

In Figures 3B, 7E, 8E multiple transwell assay images are the duplication of transwell assay images from unrelated papers of different authors, for the example - Figures 3C/3D and 7C/7D from [2] and Figures 4D and 6D from [3].

Original article: Oncotarget. 2017; 8:95880–95895. 95880-95895. https://doi.org/10.18632/oncotarget.21431
